# Sudden Caffeine Withdrawal Triggers Migraine—A Randomized Controlled Trial

**DOI:** 10.3389/fneur.2020.01002

**Published:** 2020-09-10

**Authors:** Karl B. Alstadhaug, Hilde Karen Ofte, Kai Ivar Müller, Anna P. Andreou

**Affiliations:** ^1^Nordland Hospital Trust, Bodø, Norway; ^2^Institute of Clinical Medicine, The Arctic University of Norway, Tromsø, Norway; ^3^University Hospital of Tromsø, Tromsø, Norway; ^4^Headache Research, Wolfson CARD, Institute of Psychiatry, Psychology and Neuroscience, King's College London, London, United Kingdom; ^5^The Headache Centre, Guy's and St Thomas', NHS Foundation Trust, London, United Kingdom

**Keywords:** caffeine, caffeine withdrawal, syndrome, migraine, trigger, adenosine, headache

## Abstract

**Objective:** Assessing the effects of caffeine withdrawal on migraine.

**Background:** The effects of caffeine withdrawal on migraineurs are at large unknown.

**Methods:** This was a randomized, double-blind, crossover study (NCT03022838), designed to enroll 80 adults with episodic migraine and a daily consumption of 300–800 mg caffeine. Participants substituted their estimated dietary caffeine with either placebo capsules or capsulated caffeine tablets for 5 weeks before switching the comparators for 5 more weeks.

**Results:** The study was terminated due to low recruitment. Ten subjects with a mean age of 46.3 ± 9.9 years, BMI of 24.9 ± 3.7, and a mean blood pressure of 134/83 ± 17/12 mmHg were enrolled. The average consumption of caffeine per day was 539 ± 196.3 mg. The average monthly headache days and migraine attack frequency at baseline was 11.5 ± 4.9 and 5.2 ± 1.2, respectively. At baseline Pittsburgh Sleep Quality Index was 5.8 ± 2.5 and HIT-6 was 62.8 ± 3.9. There were no differences in these or in parameters from actigraphy during the caffeine period compared with the placebo period. One subject withdrew just after entering the study. In the remaining nine, withdrawal triggered severe migraine attacks in seven, causing one more drop-out, and a typical caffeine withdrawal syndrome in two. Caffeine continuation did not trigger migraines, but one attack occurred in the wake of caffeine reintroduction.

**Conclusions:** The study failed to answer how caffeine withdrawal affects migraineurs over time, but showed that abrupt withdrawal of caffeine is a potent trigger for migraine attacks.

## Introduction

Active migraine affects more than 10% of the population, and may cause years of disability in many (1). Caffeine is the most widely psychoactive substance used (2). The effects on migraine are not fully understood, and the effects of withdrawal of caffeine on migraine has been little explored. In healthy adults, cessation of daily doses of as little as 100 mg of caffeine, the equivalent of one cup of coffee, may cause a withdrawal syndrome consisting of headaches, lethargy, and other symptoms (3). Curiously, this syndrome mimics the prodromal phase of migraine, and this seems to have been overlooked when the symptoms of caffeine withdrawal have been investigated (4). Studies explicitly documenting caffeine withdrawal as a trigger for migraine seem to be lacking.

Caffeine is strongly incriminated as a major causes of medication-overuse headache (5–7), and a strong positive correlation between caffeine consumption and both episodic (8) and chronic migraine (9) has been found in cross sectional studies. Caffeine-overuse may cause chronic headache in children (10), and the majority appears to be migraine related (11). From the findings in a retrospective population-based study, Scher et al. (12) concluded that dietary and medicinal caffeine consumption is only a modest risk factor for developing chronic daily headache, including chronic migraine. Furtehrmore, in a Norwegian large-scale cross-sectional population-based study, only a small association between high consumption of caffeine (>540 mg/d) and infrequent migraine was found (13). Chronic headache was actually associated with a low caffeine consumption (mean of 125 mg/d). In a recent prospective cohort study of adults with episodic migraine a non-linear association between caffeinated beverage intake and the odds of migraine headacahe occurrence on that day was found (14).

In this study we wanted to assess how sudden caffeine withdrawal in subjects with established episodic migraine and a regular moderate caffeine consumption would trigger migraine attack or a caffeine withdrawal syndrome. The main objective, however, was to study the “long-term” effects (headache days) on migraine. Effects on sleep and quality of life were also assessed.

## Methods

### Subjects for Inclusion

In accordance with guidelines for controlled trials of drugs in migraine by the International Headache Society Clinical Trial Subcommittee (15) adults, age 18–65, both women and men suffering from migraine for at least 1 year and fulfilling the diagnostic criteria of the International Headache Society (IHS) (16), were in general eligible for this study. Otherwise they had to be healthy, but having 3–6 attacks of migraine per month, not currently taking other medications interacting with caffeine, consuming caffeine ≥ 300 mg ≤ 800 mg every day for at least 1 month, and motivated to go through with caffeine withdrawal.

Women of fertile age had to use safe contraception, meaning barrier methods (e.g., condoms or cervical cap), hormonal methods (e.g., the pill), intrauterine devices and sterilization to ensure not getting pregnant during the study period. Conventional acute attack treatment was accepted, but not sporadic use of other drugs like hypnotics and anxiolytics. Subjects were obliged to substitute the caffeine they normally consumed with the caffeine or placebo they were provided with in the study. Even small doses of caffeine can be sufficient to suppress caffeine withdrawal headache (17), and an extra estimated average intake per day ≥25 mg in the study period was considered a breach qualifying for exclusion. Other exclusion criteria included excessive use of drugs for headache/suspicion of medication overuse headache or chronic migraine, pregnancy and breast feeding, serious co-morbidity or conditions contradicting the consumption of caffeine, working night shift, and use of drugs with moderate or major interactions with caffeine (e.g., tizanidine, lithium, phenylpropanolamine, clozapine, theophylline, adenosine, duloxetine, and ciprofloxacin). Several drugs are known to interact with caffeine, but the majority only of minor importance. All use of drugs were recorded and checked against interactions.

### Recruitment

Subjects were recruited through ordinary ambulatory clinical consultations and advertisements in the servicing area of Nordland Hospital in Bodø and The University Hospital of Tromsø. The study was also advertised through local radio. Letters to local general practitioners were also sent out.

### Enrollment and Study Procedure

Subjects who volunteered to participate received calendars (http://www.bash.org.uk/about/headache-diary/formigraine/attack/registrations), and in a 4 week run-in phase they recorded their migraine attacks. After the run-in phase study subjects came to the first visit for confirming diagnosis, clinical examination, information, and for re-evaluation of their eligibility. Pittsburgh Sleep Quality Index (PSQI) and Headache Impact Test (HIT-6) scores (see below) were obtained, and an estimate of the daily total caffeine intake (TCI) was calculated (http://www.clockworkreserach.com/caffeine-calculator/). Actigraphy motion biosensors (Actiwatch Spectrum Plus, Phillips Respironics) were worn on the wrist on the non-dominant arm and obtained data during days 3 through 7 and during days 38 through 44. If enrolled, they had to sign an informed consent form. From the point of enrollment, subjects were randomized, and then substituted their daily dietary caffeine with either capsulated placebo- or capsulated caffeine tablets (Recip®, 100 mg). The total dose (placebo or caffeine) was divided in smaller doses trying to imitate the normal pattern of dietary caffeine intake. After 5 weeks treatment was switched. Patients were informed to avoid TCI ≥ 25 mg/d in the active study period, and a risk of caffeine withdrawal symptoms within 2 days of withdrawal.

### Caffeine and Placebo Tablets

Kragerø Tablettproduksjon AS was responsible for providing caffeine tablets and producing placebo to the sponsor. The former, Koffein Recip tablets 100 mg, are approved by the Swedish medical product agency, and was imported. The placebo tablets were made identical to these. A small amount of the herb *gentiana lutea*, not known to affect headaches, was added to the placebo tablets to obtain a bitter taste similar to the caffeine tablets. Tablets were capsulated to look identical.

### Randomization and Blinding

A list of fixed allocation sequence for treatment was sent to the drug and placebo provider which used a validated system to generate a corresponding list with randomized numbers and randomized assignment of the study subjects to treatment arms in a 1:1 ratio to ensure blinding. After assignment to intervention from the list, the knowledge of the treatment was concealed from both researcher and participant.

### Meassurmenst and Outcomes

The main outcome was the mean difference in headache days between the placebo and the caffeine period. The withdrawal weeks (1 and 6) were not included.

A baseline migraine attack frequency (AF_baseline_) and migraine days (MD_baseline_) were calculated based on the calendar registrations. Registration continues during the whole study period. Patients also completed the Pittsburgh Sleep Quality Index (PSQI_baseline_) and the HIT-6 (HIT-6_baseline_) which later was repeated in week 10.

Secondary outcomes included comparison of the same arms (placebo vs. caffeine) in migraine attack frequency, PSQI scores, actigraph parameters and HIT-6 scores. The share of patients in the interventional arm that developed caffeine withdrawal or migraine syndrome was determined on the clinical evaluations 2 days after intervention (day 2 and 37).

The PSQI can be used to assess subjective sleep during the previous month (18), but is not a specific instrument for diagnosing insomnia (19). Polysomnography is the gold standard of sleep assessment, but is quite an extensive and resource intensive method. In later years, new technology has made actigraphy a reliable and easy way to measure sleep in a less invasive manner. An actigraph is a small wrist-watch-sized device that monitors movement by using an accelerometer that tracks motion and creates a graph. In addition, a button can be pushed to mark events such as bedtime or wake time. Actigraphs are widely used to study sleep-wake cycles and circadian rhythms. Several studies have compared actigraphy to polysomnography in the assessment of e.g., insomnia and other sleep disturbances, and found it to be a valid and cost-effective alternative (20–22). Total sleep, sleep latency, sleep efficacy, and the number of episodes awake during the night were used to assess sleep objectively. HIT-6 is a reliable and valid tool for discriminating headache impact across episodic and chronic migraine (23, 24).

### Assesment of Withdrawal Symptoms

Since withdrawal symptoms are considered greatest 20–48 h after the most recent intake of caffeine (25), each patient was seen by a headache specialist (KBA and KIM) on day 2 after intervention (day 2 and 37). The symptoms presented by the study subject were recorded and categorized in accordance with criteria, either as migraine (16) or (26) caffeine withdrawal.

### Statistical Analyses

The sample size was calculated based on the primary outcome measure. Meaningful response, cost vs. benefit, to caffeine withdrawal is hard to define. However, using recommended measures for clinical trials of migraine (27), a meaningful intervention is defined as a 50% reduction in headache days in 50% of the participants. To calculate the sample sizes needed to detect a difference between the two main objective binomial probabilities (success proportion in arm P1 and P2) with specified significance level and power, the following hypotheses were used: H0: P1 = P2 vs. H1: P1 ≠ P2, where P1 = caffeine withdrawal (placebo) intervention and P2 = caffeine continuation (caffeine). With significance level 95% (α = 0.05), using a one sided test on an equal sample size from two proportions, the proportion P1 = 0.5 and P2 = 0.15 are considered sufficiently different to warrant rejecting the hypothesis of no difference. Then the required sample size for two arms to achieve a 90% power (β = 0.1) could be determined by *m* (sample size arm) = 34, then *N* = 68. With a drop-out rate of 15%, 80 patients would be needed.

A paired *t*-test (two-tailed) was used to assess changes between placebo and caffeine. The Chi squared test for a 2 ×2 frequency table was applied to compare number of subjects with caffeine withdrawal syndrome with caffeine withdrawal migraine. The threshold for statistical significance was *p* < 0.05. Calculations were made by using IBM SPSS Statistics 26.

### Ethics and Study Registration

The study was conducted in accordance with *Good Clinical Practice* (28), and approved by the regional ethics committee (2015/1729REK nord) as well as by the Norwegian Medicine Agencies. It was registered at: https://www.clinicaltrials.gov/ct2/show/NCT03022838?cond=caffeine+withdrawal&rank=1.

## Results

The study started February 2017, and was terminated due to low recruitment on July 22, 2019. The exact number of subjects screened to participate was unfortunately not recorded. Just after being launched and talked about in local radio, 50 subjects volunteered through e-mail. However, it became soon clear that the majority of subjects volunteering from the community in general did not fulfill the inclusion criteria, and that the majority of subjects found eligible through ordinary clinical practice, were not willing to stop consuming dietary caffeine for 10 weeks. Only 10 subjects were enrolled and randomized, one male and nine females, with a mean age of 46.3 ± 9.9 years, an average BMI of 24.9 ± 3.7, and a mean blood pressure (BP) of 134/83 ± 17/12. The average consumption of caffeine per day was 539 ± 196.3 mg. All subjects suffered from migraine without aura, three of them also experienced some attacks with aura. Eight subjects suffered in addition from tension type headache. Two patients withdrew from the study, one due to withdrawal symptoms, and one due to potential side effects of caffeine. Table 1 summarizes the final outcomes. Overall, no significant differences were found in either headache or sleep outcomes. Table 2 summarizes the evaluations of symptoms 2 days after interventions (day 2 and 37), and the latter results are also shown as frequencies in Table 3. Comparing the proportion of subjects who experienced migraine in the wake of placebo (caffeine withdrawal) vs. caffeine (continuation or re-introduction), the Chi square with the use of Yates' correction gives: ${\text{X}}_{\text{Y}}^{2}$ = 7.244, *p* < 0.05.

**Table 1:** Headache and sleep outcome measures.

**Average number of migraine attacks** **per 4 weeks**		**Actigraphy data**	
Baseline	5.20 ± 1.22	Total sleep_Placebo_, min/night	436.00 ± 35.00
Placebo	4.89 ± 1.36	Total sleep_Caffeine_, min/night	418.22 ± 37.17
Caffeine	4.13 ± 2.10	Sleep latency_Placebo_, min/night	23.46 ± 20.50
**Days with headache per 4 weeks**		Sleep latency_Caffeine_, min/night	25.38 ± 11.83
		Awakenings_Placebo_, min/night	37.20 ± 11.84
Baseline	11.50 ± 4.88	Awakenings_Caffeine_, min/night	37.43 ± 9.35
Placebo	11.33 ± 2.45	Sleep efficacy_Placebo_,%	86.09 ± 5.09
Caffeine	12.75 ± 8.15	Sleep efficacy_Caffeine_,%	85.16 ± 3.78
**PSQI** Baseline	5.78 ± 2.49	WASO_Placebo_, min/night	32.79 ± 8.03
		WASO_Caffeine_, min/night	33.48 ± 15.66
Placebo	4.33 ± 1.50		
Caffeine	5.38 ± 1.92		
**HIT-6**		**Blood pressure,** **mmHg**	
Baseline	62.80 ± 3.91	Baseline	134/83 ± 17/12
Placebo	62.33 ± 3.84	Placebo	129/86 ± 16/13
Caffeine	62.13 ± 8.58	Caffeine	136/82 ± 21/13

*HIT-6, Headache Impact Test; PSQI, Pittsburgh Sleep Quality Index; WASO, Wake after sleep onset. All values are mean ± Std.*.

**Table 2:** Withdrawal symptoms in each study participant.

**Subject and diagnoses**	**TCI, mg**	**Symptoms in the wake of caffeine continuation**	Symptoms in the wake of caffeine discontinuation^*^	**Symptoms in the wake of caffeine re-introduction**	**Reason for study withdrawal**
1. MA, MOA, TTH	300	NA	Full-blown migraine + muscle pain in her legs	Full-blown migraine	NA
2. MOA	700	NA	Slight headache and tiredness, but in the wake of a migraine attack that started prior to intervention	“Feeling great”	NA
3. MOA, TTH	300	None	Full-blown migraine	NA	NA
4. MOA, TTH	500	NA	Caffeine withdrawal syndrome; tension type headache, tiredness, and lethargy	“Feeling okay,” but “a bit exhilarated,” nervousness and slight tremor.	NA
5. MOA, TTH	800	NA	Difficulties concentrating, dizzy, nausea, throwing up → Full-blown migraine	Palpitations and troubles with falling asleep.	NA
6. MOA, TTH	800	Rotatory vertigo, nausea, slight headache	NA	NA	Withdrew due to vertigo in the wake of caffeine continuation.
7. MOA, TTH	500	None	Full-blown migraine	NA	NA
8. MA, MOA	400	NA	Full-blown migraine with visual aura	NA	Withdrew from the study due to migraine in the wake of placebo
9. MA, MOA	800	None	Nausea, sweating, lethargy → Full-blown migraine	NA	NA
10. MOA	400	NA	Fatigue and full-blown migraine	None	NA

NA, Not applicable; None, no symptoms experienced by the patient; MA, Migraine with aura; MOA, Migraine without aura; TCI, estimated total caffeine intake; TTH, Tension type headache;

* *symptoms within 24 h*.

**Table 3:** Frequencies of migraine in the wake of placebo (caffeine withdrawal) and caffeine continuation or caffeine reintroduction.

		**Placebo**	**Caffeine**	
Migraine*	Yes	7	1	8
	No	2	7	9
	Total	9	8	17

*Comparing caffeine withdrawal (placebo) with caffeine (continuation or reintroduction) as atrigger, the Chi square with the use of Yates' correction gives a significant difference (${\text{X}}_{\text{Y}}^{2}$ = 7.244, p < 0.05).

## Discussion

Due to recruitment problems, this study failed to be completed as planned. This may reflect how higly valued caffeine is, but may also suggest that patients perceive (consciously or not) a low intake as an attack trigger. Lack of awarnes, both in society and among physicians, may also add to the problem. In the frequently cited paper of Kelman about triggers of migraine (29), caffeine withdrawal is not mentioned, perhaps because of the pharing in the questionnaire. In some reviews, however, it is considered as a quite common trigger (30), but solid documentation for this has been lacking.

A regular use of caffeine equivalent to the amount consumed by the participants in this study has been estimated to occupy up to 50% of adenosine receptors in the brain (31). Evidence supports that adenosine receptors are involved in headache pathophysiology (32), including migraine (4). Abrupt caffeine withdrawal may cause rebound headache, a widely known phenomenon, an well-documented also in randomized controlled studies (3), but whether it triggers migraine is largely unknown. In an observational study of 32 migraineurs before and during Ramadan fasting, the number of migraine days increased in average from 3.7 ± 2.1 to 9.4 ± 4.3 (*p* < 0.001). The negative effect was attributed to dehydration and caffeine withdrawal, but this was not specifically assessed (33). Couturier et al. (34) suggested that caffeine withdrawal could be related to weekend migraine. In the present study caffeine withdrawal triggered severe migraine attacks in seven out of nine. Caffeine continuation did not trigger migraine in any of the study subjects, but one attack occurred in the wake of caffeine reintroduction. Unfortunately, due to the study protocol, triggered attacks could not be treated with caffeine.

In a recent study, showing that caffeine does not affect the susceptibility to cortical spreading depolarization, the phenomenon considered responsible for the migraine aura, the investigators conclude that the adverse or beneficial effects of caffeine on headache seem unrelated to CSD pathophysiology, consistent with the non-migrainous clinical presentation of caffeine-related headache (35). Our study strongly indicates that abrupt caffeine withdrawal in patients with migraine actually triggers migraine. This was also noted in the early description of caffeine-withdrawal headache by Dreisbach (36); in migraineurs typical migraine syndromes ensued. Coturier et al. (34) linked weekend attacks of migraine to caffeine withdrawal, but did not assess this specifically. All of the three migraineurs with aura in our study got attacks triggered by caffeine withdrawal, but only one attack was with aura.

The primary objective of this study was to determine how caffeine withdrawal over 5 weeks would affect the migraine burden. The long-term effect of caffeine withdrawal is little studied. Hering-Hanit and Gadoth reported 36 children with chronic headache, none with a prior history of migraine, who consumed excessive amounts of caffeine in the form of cola drinks (in average ~190 mg caffeine/d), and of whom 33 completely recovered after gradual withdrawal. Three children continued to have infrequent migraine without aura after withdrawal (10). In a double blinded, randomized, placebo-controlled, 12 week crossover study of 45 healthy subjects who habitually consumed 4–6 cups of coffee a day, withdrawal caused headaches during the first or second day in 19 (42%) (37). Subjects reporting headaches during caffeine vs. non-caffeine weeks were similar (ratio 1.1). However, what is obvious, but not commented by the authors, is that when the withdrawal weeks (week 1 and 7) are removed the ratio increases to 7.7. Despite small numbers, the result indicates that long-term elimination of caffeine may be beneficial in reducing headache burden. Subjects also reported improved sleep during the non-caffeine weeks. Due to the low number of participants, the present study failed to demonstrate any long-term consequences of detoxification. However, both fewer days with headache and lower PSQI during the placebo period when compared to the caffeine period were observed.

As suggested by Spencer (38), caffeine withdrawal in migraineurs may serve as a human model for studying migraine. Why withdrawal could act as a trigger is unknown, but it is probably a consequence of an up-regulation of adenosine receptors during regular caffeine intake (4). Increased sensitivity to adenosine after withdrawal may increase excitability in networks involved in migraine pathophysiology, lowering the threshold for attacks. Fried et al. (32) suggested that it is the adenosine receptor A2A signaling that contribute to headache via modulation of intracellular cyclic adenosine monophosphate (cAMP) in neurons and glia to subsequently affect glutaminergic synaptic signaling. Based on an *in vivo* rat model of migraine, (39) suggested that A2A receptor antagonism may offer a novel approach to antimigraine therapy. Imaging studies using positron emission tomography (PET) A1AR- (31) and A2AR-ligands are suitable for studying the cerebral effects of caffeine (40), and may perhaps also be suitable for studying the effects of caffeine withdrawal as a trigger for migraine *in vivo*.

In conclusion, this study offers evidence on the acute trigger of migraine attacks following caffeine in migraine patients. Larger studies are needed to vaildate the finding.

## Data Availability Statement

The raw data supporting the conclusions of this article will be made available by the authors, without undue reservation.

## Ethics Statement

The studies involving human participants were reviewed and approved by the Regional Ethics Committee (2015/1729REK nord). The patients/participants provided their written informed consent to participate in this study.

## Author Contributions

KA designed and conceptualized the study, acquired data, analyzed and interpreted them, and drafted the manuscript. HO helped designing the study and revised the manuscript for intellectual content. KM acquired data and revised the manuscript for intellectual content. AA helped designing the study and revised the manuscript for intellectual content. All authors contributed to the article and approved the submitted version.

## Conflict of Interest

The authors declare that the research was conducted in the absence of any commercial or financial relationships that could be construed as a potential conflict of interest.

## References

[1] GBD 2017 Disease and Injury Incidence and Prevalence Collaborators Global, regional, and national incidence, prevalence, and years lived with disability for 354 diseases and injuries for 195 countries and territories, 1990-2017: a systematic analysis for the Global Burden of Disease Study 2017. Lancet. (2018) 392:1789–858. 10.1016/S0140-6736(18)32279-7 30496104 PMC6227754

[2] Fredholm BB Battig K Holmen J Nehlig A Zvartau EE. Actions of caffeine in the brain with special reference to factors that contribute to its widespread use. Pharmacol Rev. (1999) 51:83–133. 10049999

[3] Silverman K Evans SM Strain EC Griffiths RR. Withdrawal syndrome after the double-blind cessation of caffeine consumption. N Engl J Med. (1992) 327:1109–14. 10.1056/NEJM199210153271601 1528206

[4] Alstadhaug KB Andreou AP Caffeine and primary (Migraine) headaches-friend or foe? Front Neurol. (2019) 10:1275 10.3389/fneur.2019.01275 31849829 PMC6901704

[5] Diener HC Limmroth V. Medication-overuse headache: a worldwide problem. Lancet Neurol. (2004) 3:475–83. 10.1016/S1474-4422(04)00824-5 15261608

[6] Dong Z Chen X Steiner TJ Hou L Di H He M. Medication-overuse headache in China: Clinical profile, and an evaluation of the ICHD-3 beta diagnostic criteria. Cephalalgia. (2014) 35:644–51. 10.1177/0333102414552533 25286910

[7] Scher AI Lipton RB Stewart WF Bigal M. Patterns of medication use by chronic and episodic headache sufferers in the general population: results from the frequent headache epidemiology study. Cephalalgia. (2010) 30:321–8. 10.1111/j.1468-2982.2009.01913.x 19614708

[8] Takeshima T Ishizaki K Fukuhara Y Ijiri T Kusumi M Wakutani Y. Population-based door-to-door survey of migraine in Japan: the Daisen study. Headache. (2004) 44:8–19. 10.1111/j.1526-4610.2004.04004.x 14979878

[9] Bigal ME Sheftell FD Rapoport AM Tepper SJ Lipton RB. Chronic daily headache: identification of factors associated with induction and transformation. Headache. (2002) 42:575–81. 10.1046/j.1526-4610.2002.02143.x 12482208

[10] Hering-Hanit R Gadoth N. Caffeine-induced headache in children and adolescents. Cephalalgia. (2003) 23:332–5. 10.1046/j.1468-2982.2003.00576.x 12780761

[11] Hershey AD. Chronic daily headaches in children. Expert Opin Pharmacother. (2003) 4:485–91. 10.1517/14656566.4.4.485 12667111

[12] Scher AI Stewart WF Lipton RB. Caffeine as a risk factor for chronic daily headache: a population-based study. Neurology. (2004) 63:2022–7. 10.1212/01.WNL.0000145760.37852.ED 15596744

[13] Hagen K Thoresen K Stovner LJ Zwart JA. High dietary caffeine consumption is associated with a modest increase in headache prevalence: results from the Head-HUNT Study. J Headache Pain. (2009) 10:153–9. 10.1007/s10194-009-0114-6 19308315 PMC3451984

[14] Mostofsky E Mittleman MA Buettner C Li W Bertisch SM. Prospective cohort study of caffeinated beverage intake as a potential trigger of headaches among migraineurs. Am J Med. (2019) 132:984–91. 10.1016/j.amjmed.2019.02.015 31402050 PMC6744320

[15] Tfelt-Hansen P Pascual J Ramadan N Dahlof C D'Amico D Diener HC. Guidelines for controlled trials of drugs in migraine: third edition. A guide for investigators. Cephalalgia. (2012) 32:6–38. 10.1177/0333102411417901 22384463

[16] Headache Classification Committee of the International Headache Society (IHS) The international classification of headache disorders, 3rd edition (beta version). Cephalalgia. (2013) 33:629–808. 10.1177/0333102413485658 23771276

[17] Griffiths RR Reissig CJ Substance Abuse: Caffeine Use Disorders. Psychiatry: John Wiley & Sons, Ltd (2008). p. 1019–40. 10.1002/9780470515167.ch56

[18] Buysse DJ Reynolds CF III Monk TH Berman SR Kupfer DJ. The Pittsburgh sleep quality index: a new instrument for psychiatric practice and research. Psychiatry Res. (1989) 28:193–213. 10.1016/0165-1781(89)90047-4 2748771

[19] Riemann D Baglioni C Bassetti C Bjorvatn B Dolenc Groselj L Ellis JG. European guideline for the diagnosis and treatment of insomnia. J Sleep Res. (2017) 26:675–700. 10.1111/jsr.12594 28875581

[20] McCall C McCall WV. Comparison of actigraphy with polysomnography and sleep logs in depressed insomniacs. J Sleep Res. (2012) 21:122–7. 10.1111/j.1365-2869.2011.00917.x 21447050 PMC3134551

[21] Lichstein KL Stone KC Donaldson J Nau SD Soeffing JP Murray D. Actigraphy validation with insomnia. Sleep. (2006) 29:232–9. 16494091

[22] Natale V Plazzi G Martoni M. Actigraphy in the assessment of insomnia: a quantitative approach. Sleep. (2009) 32:767–71. 10.1093/sleep/32.6.767 19544753 PMC2690564

[23] Yang M Rendas-Baum R Varon SF Kosinski M. Validation of the headache impact test (HIT-6) across episodic and chronic migraine. Cephalalgia. (2011) 31:357–67. 10.1177/0333102410379890 20819842 PMC3057423

[24] Kosinski M Bayliss MS Bjorner JB Ware JE Jr Garber WH Batenhorst A. A six-item short-form survey for measuring headache impact: the HIT-6. Qual Life Res. (2003) 12:963–74. 10.1023/A:1026119331193 14651415

[25] Griffiths RR Woodson PP. Caffeine physical dependence: a review of human and laboratory animal studies. Psychopharmacology. (1988) 94:437–51. 10.1007/BF00212836 3131789

[26] American Psychiatric Association APADSMTF Diagnostic and Statistical Manual of Mental Disorders: DSM-5. Washington, DC: American Psychiatric Association (2013). 10.1176/appi.books.9780890425596

[27] Olesen J Krabbe AA Tfelt-Hansen P. Methodological aspects of prophylactic drug trials in migraine. Cephalalgia. (1981) 1:127–41. 10.1046/j.1468-2982.1981.0103127.x 7346180

[28] Agency EM Note for Guidance on Good Clinical Practice. (2016). Available online at: http://www.ema.europa.eu/docs/en_GB/document_library/Scientific_guideline/2009/09/WC500002874.pdf (accessed August 27, 2020).

[29] Kelman L The triggers or precipitants of the acute migraine attack. Cephalalgia. (2007) 27:394–402. 10.1111/j.1468-2982.2007.01303.x 17403039

[30] Marmura MJ. Triggers, protectors, and predictors in episodic migraine. Curr Pain Headache Rep. (2018) 22:81. 10.1007/s11916-018-0734-0 30291562

[31] Elmenhorst D Meyer PT Matusch A Winz OH Bauer A. Caffeine occupancy of human cerebral A1 adenosine receptors: *in vivo* quantification with 18F-CPFPX and PET. J Nucl Med. (2012) 53:1723–9. 10.2967/jnumed.112.105114 22966134

[32] Fried NT Elliott MB Oshinsky ML. The role of adenosine signaling in headache: a review. Brain Sci. (2017) 7:30. 10.3390/brainsci7030030 28335379 PMC5366829

[33] Abu-Salameh I Plakht Y Ifergane G. Migraine exacerbation during Ramadan fasting. J Headache Pain. (2010) 11:513–7. 10.1007/s10194-010-0242-z 20652352 PMC3476230

[34] Couturier EG Hering R Steiner TJ. Weekend attacks in migraine patients: caused by caffeine withdrawal? Cephalalgia. (1992) 12:99–100. 10.1046/j.1468-2982.1992.1202099.x 1576651

[35] Yalcin N Chen SP Yu ES Liu TT Yen JC Atalay YB Caffeine does not affect susceptibility to cortical spreading depolarization in mice. J Cereb Blood Flow Metab. (2019) 39:740–50. 10.1177/0271678X18768955 29651899 PMC6446422

[36] The Scientific Proceedings of the American Society for Pharmacology and Experimental Therapeutics Incorporated: Thirty-First Annual Meeting New Orleans Louisiana March 13–16 1940 J Pharmacol Exp Ther. (1940) 69:273–310.

[37] van Dusseldorp M Katan MB. Headache caused by caffeine withdrawal among moderate coffee drinkers switched from ordinary to decaffeinated coffee: a 12 week double blind trial. BMJ. (1990) 300:1558–9. 10.1136/bmj.300.6739.1558 2372623 PMC1663116

[38] Spencer B. Caffeine withdrawal: a model for migraine? Headache. (2002) 42:561–2. 10.1046/j.1526-4610.2002.02137.x 12167153

[39] Haanes KA Labastida-Ramírez A Chan KY de Vries R Shook B Jackson P. Characterization of the trigeminovascular actions of several adenosine A 2A receptor antagonists in an in vivo rat model of migraine. J Headache Pain. (2018) 19:41. 10.1186/s10194-018-0867-x 29802484 PMC5970128

[40] Mishina M Ishiwata K. Adenosine receptor PET imaging in human brain. Int Rev Neurobiol. (2014) 119:51–69. 10.1016/B978-0-12-801022-8.00002-7 25175960

